# The bared external anal sphincter (BEAS), a new technique for high horseshoe anal fistula: a hospital-based cohort study

**DOI:** 10.1038/s41598-023-32698-y

**Published:** 2023-04-11

**Authors:** Jingyi Zhu, Peixin Du, Zhenyi Wang, De Zheng, Qingming Wang, Zubing Mei

**Affiliations:** 1 Department of Anorectal Surgery, Yueyang Hospital of Integrated Chinese and Western Medicine, Affiliated to Shanghai University of Traditional Chinese Medicine, Shanghai, China; 2 Department of Anorectal Surgery, Shuguang Hospital Affiliated to Shanghai University of Traditional Chinese Medicine, 528 Zhangheng Road, Shanghai, 201203 China; 3 Anorectal Disease Institute of Shuguang Hospital, Shanghai, China

**Keywords:** Colorectal surgery, Outcomes research

## Abstract

The aim of this study was to introduce a new technique, the bared external anal sphincter technique, and to evaluate its effectiveness and safety for primary or recurrent high horseshoe anal fistula (HHAF). We used data from a tertiary referral hospital’s prospective database of a hospital-based cohort. All the patients underwent the bared external anal sphincter procedure. The main outcomes were short‐term clinical outcomes including the 6-month cure rate, Visual Analog Scale pain score (VAS-PS) and Cleveland Clinic Florida incontinence score (CCF-IS). The secondary outcomes included the Quality of Life in Patients with Anal Fistula Questionnaire score (QoLAF-QS), Bristol stool chart and postoperative complications. A total of 48 HHAF patients (39 males) with a mean age of 34.2 years (SD 9.04; range, 21–54) were analyzed in this retrospective study. At the 6-month follow-up, the average VAS-PS and CCF-IS were 0.81 (SD 2.28; range, 0–10) and 1.29 (SD 2.87; range, 0–13), respectively. QoLAF-QS showed that the bared external anal sphincter procedure had no impact over their quality of life in 45 patients (93.75%), limited impact in 2 patients (4.16%), and moderate impact in one patient (2.08%). The Bristol stool scale showed that all patients had normal stool characteristics. The 6-month cure rate was 93.75%. Three patients (6.25%) experienced recurrent symptoms but recovered after surgical management. Urinary retention occurred in 1 case (2.78%). No other postoperative complications were reported. No patient had anal incontinence. The bared external anal sphincter procedure is a safe, effective and sphincter-sparing approach for patients with primary or recurrent HHAF in terms of short‐term results.

## Introduction

High horseshoe anal fistula (HHAF) is a refractory anorectal disease. The reported incidence of all anal fistula is approximately 8.6 per 100,000 people, and HHAF accounts for approximately 2–5% of all anal fistulas^1,2^. The primary internal opening of the HHAF is often located at the posterior midline line (66.4%), which often involves the deep intersphincteric space (DPIS, 75%) and the deep postanal space (DPAS, 25%), leading to a high rate of postoperative recurrence and anal incontinence^3^. Fistulotomy, cutting seton, and modified Hanley procedure have long been used to treat HHAF^4^. Although these procedures have high cure rates, they could also cause damage to the anal sphincter, leading to the increased risk of fecal incontinence^5,6^.

In recent years, new procedures, including the anal fistula plug, fibrin glue injection, ligation of the intersphincteric fistula tract (LIFT), endorectal advancement flap (ERAF), video-assisted anal fistula treatment (VAAFT), laser ablation of the fistula tract (LAFT) and fistula laser closure (FiLaC®) technique, have been shown to be relatively safe but have low cure rates for HHAF^7–13^. Nevertheless, there have been few clinical reports on the treatment of HHAF. As a result, developing an effective and mature surgical technique for the treatment of HHAF remains a significant challenge. Herein, we reported a novel surgical procedure, the bared external anal sphincter (BEAS) technique, to treat HHAF with the goal of closing the internal opening of the anal fistula, preserving sphincter function, and reducing the original wound size. We designed this study to retrospectively analyze prospective data to assess the efficacy and safety of BEAS.

## Methods and analysis

### Study design and population

The study was a retrospective analysis of prospectively collected data from a cohort from the tertiary referral center (Shuguang Hospital). Consecutive adult patients diagnosed with HHAF undergoing BEAS technique between June 2020 and January 2021 were included. Ethical approval was obtained from the ethics committee of Shuguang Hospital Affiliated with Shanghai University of Traditional Chinese Medicine (Approval No. 2020-823-30-01). Written informed consent was obtained from each participant. All methods were carried out in accordance with relevant guidelines and regulations.

### Inclusion and exclusion criteria

Magnetic resonance imaging (MRI) was performed on every patient, which helped to determine the extent of the HHAF lesion and its relationship with surrounding tissues. The diagnosis of HHAF was made and confirmed by at least two senior imaging specialists.

The inclusion criteria were the following: (1) male or female patients aged 18 to 65 years; and (2) patients diagnosed with high cryptoglandular fistula-in-ano (involving more than one-third of the sphincter complex as assessed on MRI and intraoperative examination under anesthesia). Both primary and recurrent horseshoe fistulas were included. Patients with Crohn's disease, cancer, tuberculosis, diabetes, autoimmune diseases or patients receiving long-term steroids or corticosteroid therapy were excluded.

### Follow-up and outcome measures

Patient demographics, clinical information, and short-term clinical outcome data were collected through outpatient follow-up, a WeChat questionnaire and telephone follow-up. Forty-one patients were followed-up by WeChat questionnaire and seven patients were followed-up by phone. There is no difference between these methods. The main outcomes included the 6-month cure rate, Visual Analog Scale pain score (VAS-PS) and Cleveland Clinic Florida incontinence score (CCF-IS). The secondary outcomes included the Quality of Life in Patients with Anal Fistula Questionnaire score (QoLAF-QS), Bristol stool chart and postoperative complications. Postoperative pain was measured using an 11-point Visual Analog Scale pain score (VAS-PS)^14^. The severity of fecal incontinence symptoms was evaluated using the Cleveland Clinic Florida incontinence score (CCF-IS)^15^. The Quality of Life in Patients with Anal Fistula Questionnaire score (QoLAF-QS) was used to assess the quality of life of patients with anal fistula^16^. Stool consistency was assessed using the 7-point Bristol stool scale^17^. Disease recurrence, as was reported by Mei et al., was defined as persistence or recurrence of symptoms or the relapse of the perianal sepsis within or more than 6 months following surgical intervention^18,19^.

### Statistical analysis

SPSS Statistics 25.0 (IBM Inc., IL, USA) software was used for ststistical analysis. Continuous variables are presented as the mean ± standard deviation (SD) or median with interquartile range (IQR) based on distribution. The independent t test was used to compare normally distributed continuous variables, and the Mann–Whitney U test was used to compare nonnormally distributed continuous variables. Categorical data are expressed as the number of cases and percentages.* P* < 0.05 was considered to indicate a ststistically significant difference.

### Operative technique

#### Preparation for Surgery

**Figure 1:**
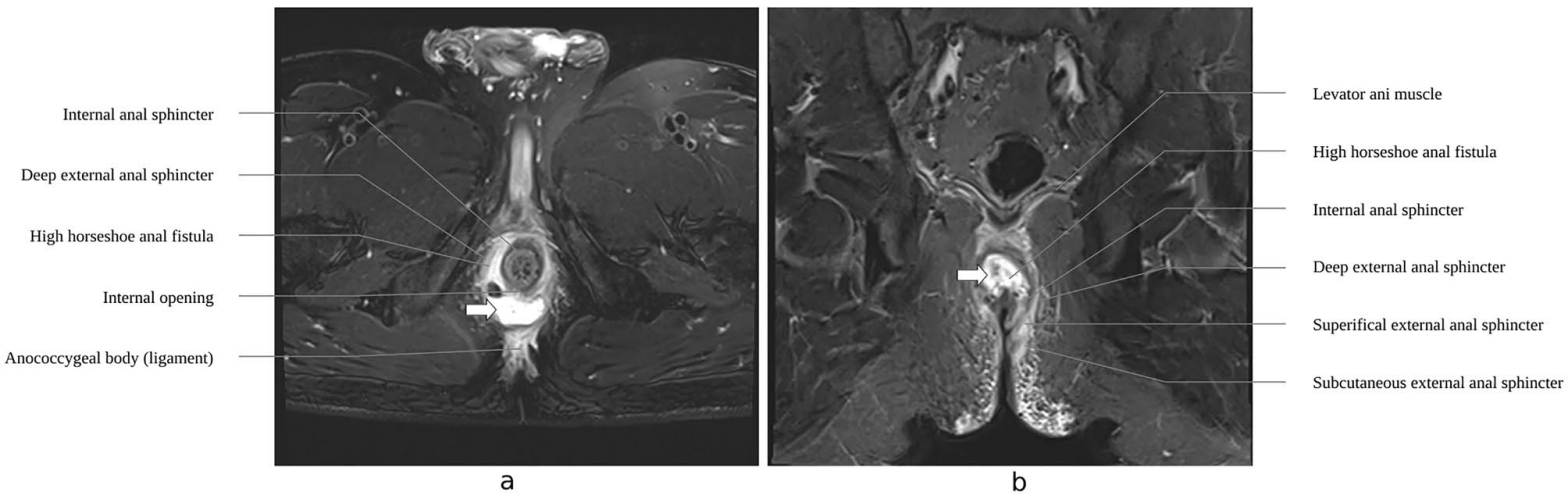
The diagrams of preoperative MRI. (**a**) The cross section of the perianal structure showing the relationship between IAS, EAS and HHAF. (**b**) The coronal section of the pelvis showing layers of anal sphincter, especially the levator ani muscle, and HHAF. IAS = internal anal sphincter; EAS = external anal sphincter; HHAF = high horseshoe anal fistula.

Preparation for surgery begins with a careful evaluation of preoperative MRI to assess the location of the internal opening and the extent of inflammation as well as the relationship between the fistula and the muscles. The imaging also informs about the anatomical structure of anal canal, aiding in operative planning (Fig. 1).

#### Setup and exposure

**Figure 2:**
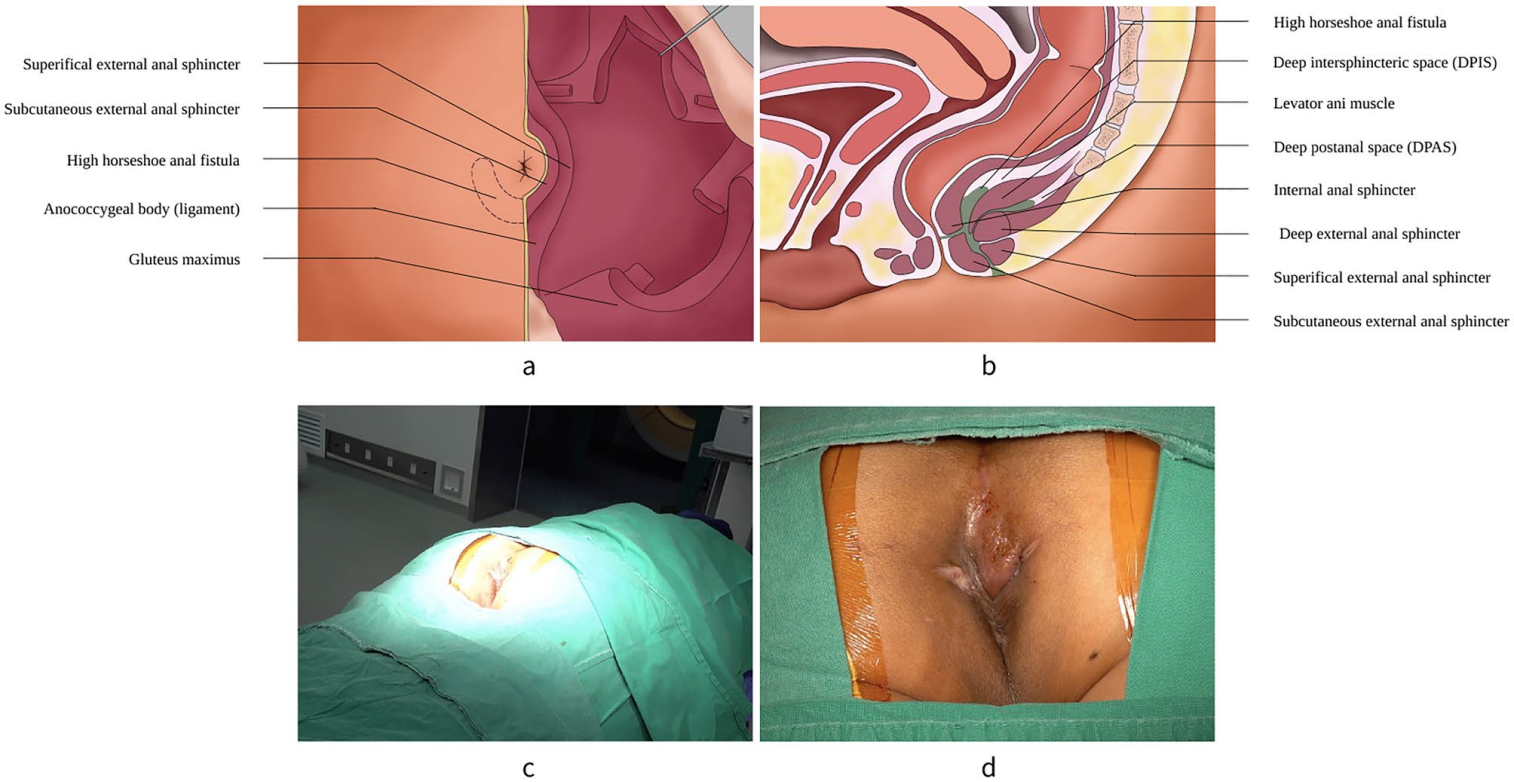
Anatomic Structure of HHAF. (**a**) View of the outside appearance. The dotted line represents the scope HHAF. (**b**) Sagittal section of the pelvis. (**c**) Schematic diagram of posture for surgical exposure. (**d**) Preoperative visual field. The green shaded part represents HHAF. HHAF = high horseshoe anal fistula.

The patient is given spinal anesthesia and then placed in prone jackknife position. After preparing and draping, the operating table is placed in a 10° to 15° head-side-down position. This allows the muscles and spaces exposed more clearly in posterior aspect of anal canal during the operation. The internal opening, the external opening and the fistula of HHAF is then identified again to begin dissection (Fig. 2).

### Step 1: Intersphincteric Approach (IS Approach)

**Figure 3:**
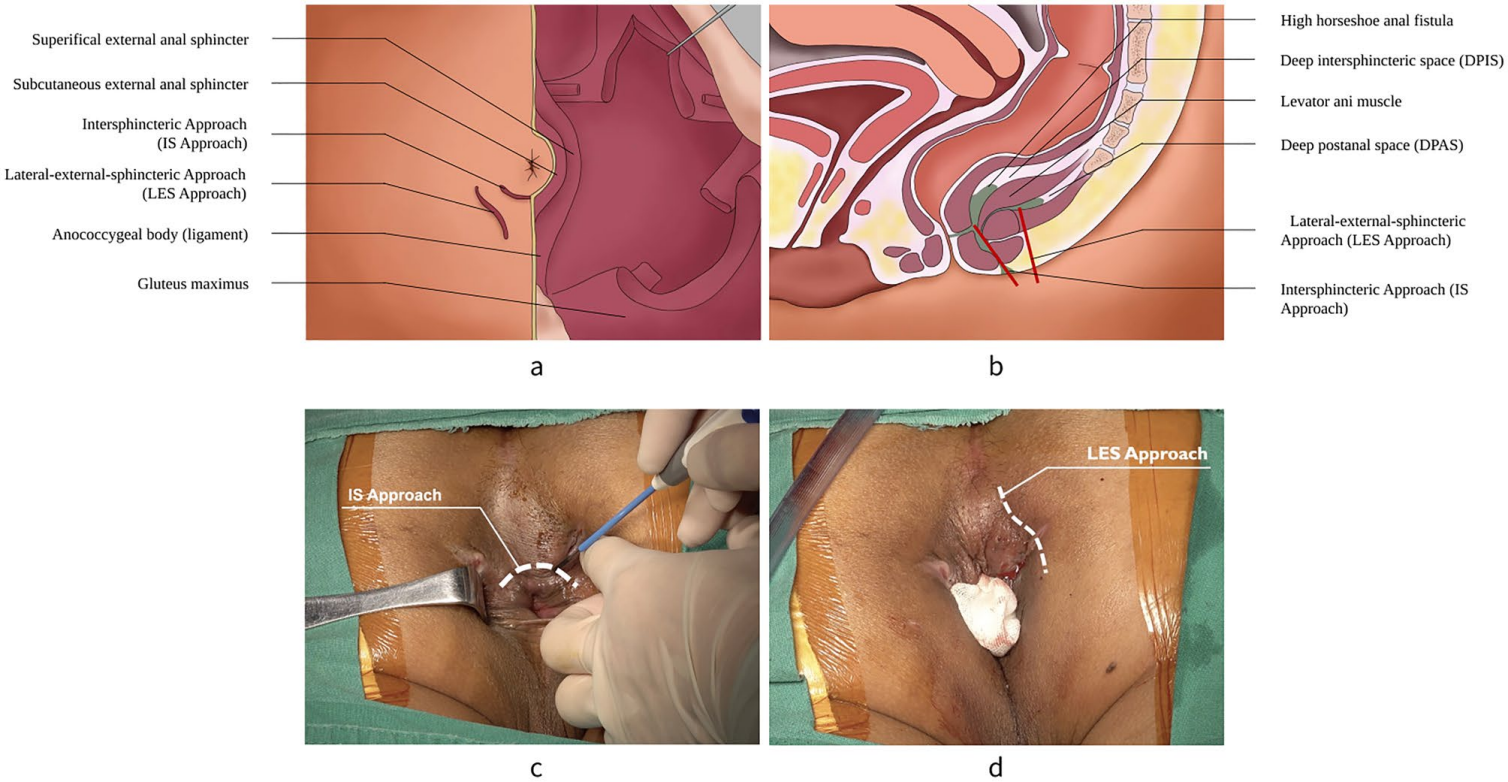
The operation diagram of IS approach and LES approach. (**a**) View of the outside appearance. (**b**) Sagittal section of the pelvis. The dissection of IS approach is along the intersphincteric plane to separate the IAS from EAS. (**c**) IS approach. (**d**) LES approach. The dissection of LES approach is along the outer edge of the EAS to bare the EAS. IS = Intersphincteric; IAS = internal anal sphincter; EAS = external anal sphincter; LES = Lateral-external-sphincteric.

The dissection is initiated with a curvilinear incision (IS approach) along the intersphincteric groove to identify the internal anal sphincter (IAS) and external anal sphincter (EAS). This incision is directly behind the anal canal, which is approximately 1/4–1/3 of a quadrant of the anus. Then, the dissection is performed along the plane of the intersphincteric groove to separate the IAS from EAS with an electrical scalpel. The internal opening should be concerned during the dissections. Through both the anal canal and intersphincteric plane, the internal opening can be identified easily. There is barely no blood supply in the intersphincteric plane, therefore it is a safe dissection plane. However, care should be taken to observe the muscle contraction of EAS during this dissection. Because dissection is close to the IAS and EAS, the surgeon should take care during the dissection to avoid inadvertent injury. To avoid complications of incontinence or bleeding, the surgeon should dissect the IAS and EAS strictly along the plane (Fig. 3).

### Step 2: Lateral-external-sphincteric approach (LES approach)

The next step involves the dissection of the EAS, which is initiated with a curvilinear incision (LES approach) along the outer edge of the EAS on one side behind the anal canal. The dissection is performed along the outer edge of the EAS until above the level of the deep EAS so as to bare the EAS. The lateral part of the EAS in the corresponding quadrant is exposed with the traction of a self-retaining retractor (Lone Star, Cooper Surgical, Trumbull, CT). The highest risk for incontinence, which is the most common postoperative complication, may be due to the injury of EAS. The bareness of EAS can completely expose the infection focus of HHAF. In this process, the surgeon should also be mindful of avoiding the anterior displacement of anal canal caused by the injury of anococcygeal ligament (Fig. 3).

### Step 3: Exposure of DPIS

**Figure 4:**
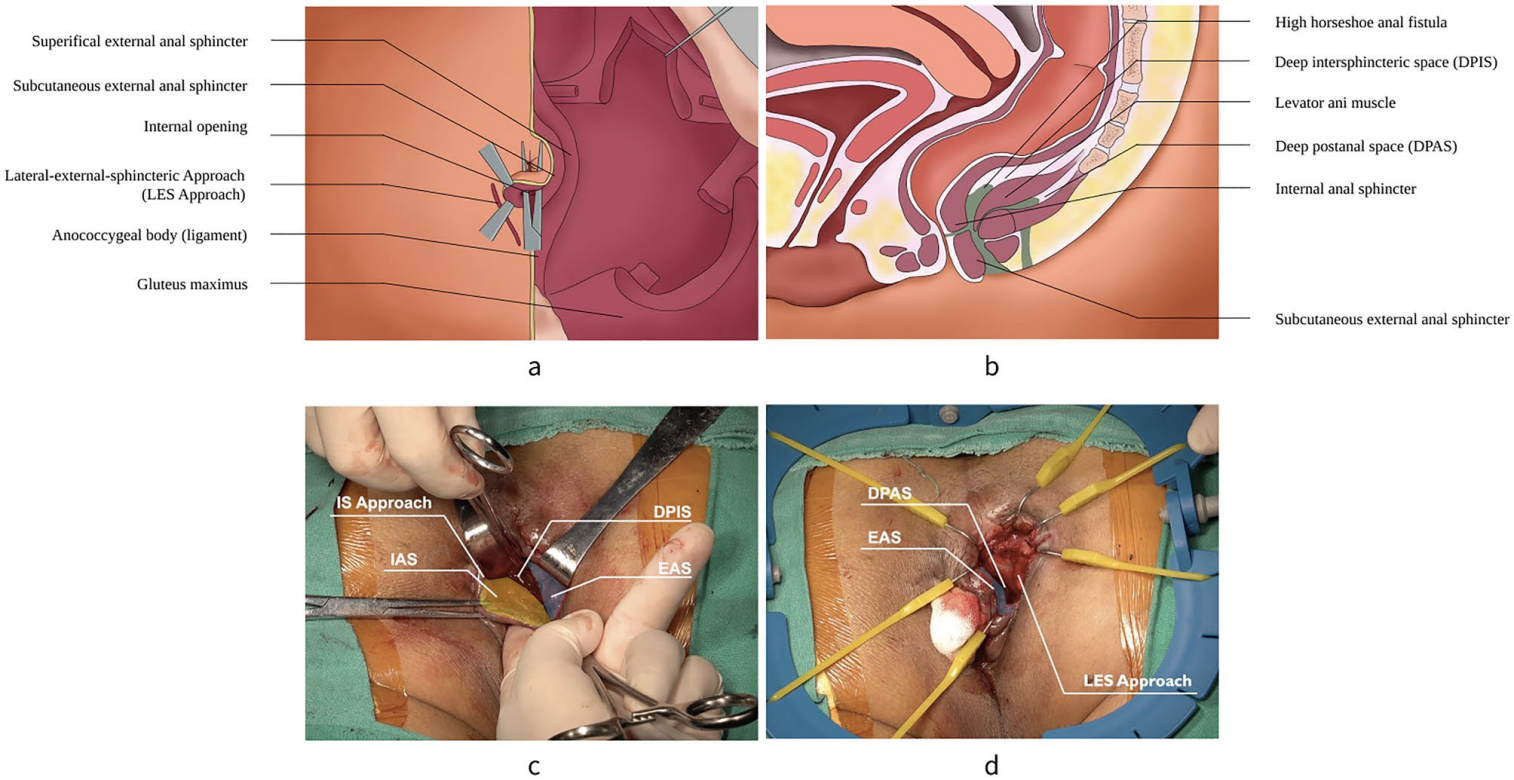
The operation diagram of exposure of DPIS and DPAS. (**a**) View of the outside appearance. (**b**) Sagittal section of the pelvis. (**c**) Exposure of DPIS. (**d**) Exposure of DPAS. Expose DPIS and DPAS to reach the fistula through IS approach and LES approach, respectively. DPIS = deep intersphincteric space; DPAS = deep postanal space.

Once the IAS and EAS are separated, medial to lateral dissection of the muscles are continued along the intersphincteric plane to both sides. Then, the IAS is separated from EAS by a combination of sharp and blunt dissection. Through the IS approach, the suprasphincter anal fistula can be detected above the level of the deep EAS easily. Cephalad dissection is continued above or beneath the levator ani muscle so that the DPIS and the inner part of the EAS could be completely exposed (Fig. 4).

### Step 4: Exposure of DPAS

Continuing the dissection cephalad with the assist of self-retaining retractor along the LES approach reveals the DPAS, which can then be handled at the top of the infection. Both two approaches communicate at the top of the EAS (or at the top point of the pus cavity of the HHAF). Typically, the visualization of these approaches reveals the pus cavity under direct vision. The aim of these dissections is to utilize both the IS approach and the LES approach as a landmark to ensure a complete preservation of the EAS (Fig. 4).

### Step 5: Musculomucosal flap and EAS advancement

**Figure 5:**
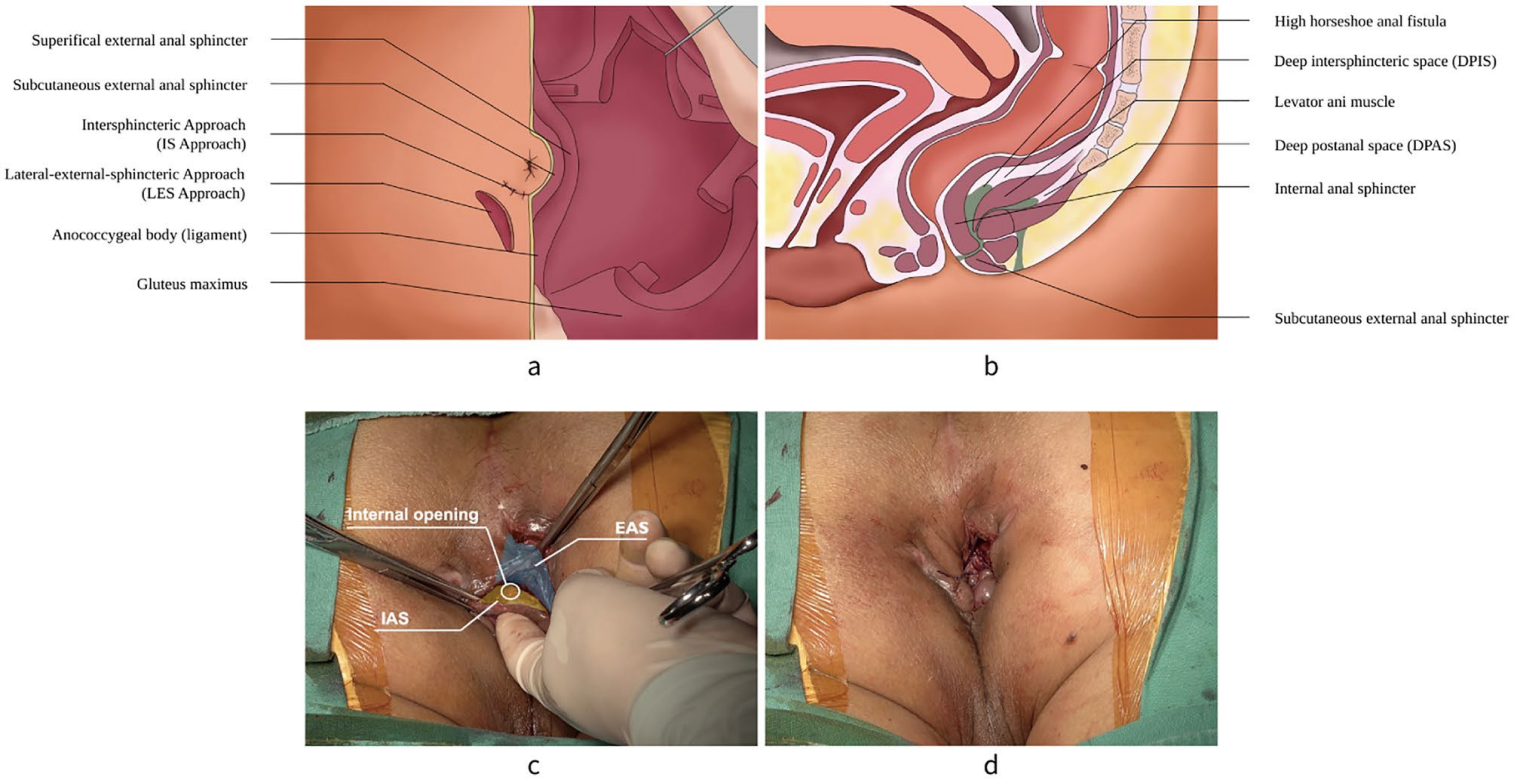
The operation diagram of musculomucosal flap and EAS advancement. (**a**) View of the outside appearance. (**b**) Sagittal section of the pelvis. (**c**) Musculomucosal Flap and EAS Advancement. (**d**) Visual field after suture. Perform advancement of the musculomucosal flap and the EAS to confirm the internal opening could reach the inferior edge of the EAS without tension. Then close the intersphincteric incision (IS approach) in an interrupted manner and keep LES approach. EAS = external anal sphincter; IS = Intersphincteric; LES = Lateral-external-sphincteric.

After the DPIS, the DPAS, and the pus cavity are irrigated repeatedly with povidone and hydrogen peroxide, the bare EAS is pushed proximally to confirm that the internal opening on the musculomucosal flap could reach the inferior edge of the EAS without tension. After the musculomucosal flap and the EAS advancement are performed, they are sutured and fixed with 2–0 Polyglactin suture (Coated VICRYL, 2–0, ETHICON Inc, China) to close the intersphincteric incision in an interrupted manner. At last, the LES approach is kept open and indwelled with povidone gauze to facilitate postoperative drainage (Fig. 5).

## Results

A total of 48 HHAF patients with a mean age of 34.2 years were treated with BEAS. The fistulas were suprasphincteric (87.50%), extrasphincteric (8.33%) and high intersphincteric (4.17%). A total of 18.75% fistulas invaded the DPIS, 16.67% invaded the DPAS and 64.58% invaded both the DPIS and DPAS (Table 1). Most patients (41/48) had posterior HHAF, 4 had anterior HHAF, and 3 had circular HHAF (Table 1). Table 2 presents the baseline characteristics of the included patients, among which 31 patients were primary HHAF and 17 patients were recurrent ones. All the 3 patents who developed postoperative recurrence were from the 17 recurrent patients, who were classified as supra-sphincter based on the Park's classification of perianal fistulas. Among these 3 recurrent fistulas, one was circular HHAF and 2 were posterior HHAFs.

**Table 1:** Fistula characteristics.

Variables	Patients (N = 48)
Symptoms, n (%)	
None	11 (22.92)
Purulent	24 (50.00)
Pain	31 (64.58)
Anal bleeding	10 (20.83)
Anal itching	26 (54.17)
Parks classification, n (%)	
Suprasphincter fistula	42 (87.50)
Extrasphincter fistula	4 (8.33)
High intersphincteric fistula	2 (4.17)
Types of horseshoe anal fistula, n (%)	
Circular	3 (6.25)
Anterior	4 (8.33)
Posterior	41 (85.42)
Location of internal opening, n (%)	
Anterior	4 (8.33)
Posterior	44 (91.67)
Distance between internal opening and anal margin, cm (mean ± SD)	3.25 ± 0.80
Space involved, n (%)	
DPIS	9 (18.75)
DPAS	8 (16.67)
DPIS and DPAS	31 (64.58)

DPIS, deep intersphincteric space; DPAS, deep postanal space.

**Table 2:** Patient demographics and characteristics.

Variables	Patients (N = 48)
Sex, n (%)	
Female	9 (18.75)
Male	39 (81.25)
Age, y (mean ± SD)	35.10 ± 9.04
Height, m (mean ± SD)	1.73 ± 0.08
Weight, kg (mean ± SD)	78.65 ± 21.28
BMI, kg/m^2^ (mean ± SD)	25.44 ± 8.23
Comorbidities, n (%)	
None	46 (95.83)
Diabetes mellitus	2 (4.17)
Hypertension	1 (2.08)
Other pathology	0 (0)
Recurrence history, n (%)	
No	31 (64.58)
Yes	17 (35.42)
Prior anal surgery, n (%)	
No	31 (64.58)
Yes	17 (35.42)
Prior abscess drainage, n (%)	
No	35 (72.92)
Yes	13 (27.08)
Tertiary referral, n (%)	
No	3 (6.25)
Yes	45 (93.75)
Smoking use, n (%)	
No	22 (45.83)
Yes	26 (54.17)
Alcohol use, n (%)	
No	28 (58.33)
Yes	20 (41.67)

BMI, body mass index.

We present the main results of our study in Tables 3 and 4. In brief, during the mean follow-up time of 11.3 months (SD 3.87; range, 6–21), 45 patients (93.75%) recovered after the BEAS procedure. Three patients (6.25%) experienced recurrent disease during the follow-up period. One had impaired healing of the internal opening at 7 weeks after surgery and recovered after transanal opening of intersphincteric space (TROPIS) procedure. The other two patients had poor wound drainage after surgery at 9 and 13 weeks after surgery, and both recovered well after a second BEAS procedure. Urinary retention occurred in 1 case (2.78%). No other postoperative complications were reported.

**Table 3:** Operation data and clinical outcomes.

Variables	Patients (N = 48)
Bowel preparation, n (%)	
None	5 (10.42)
Enema (s)	40 (83.33)
Mechanical preparation	3 (6.25)
ASA score, n (%)	
I	46 (95.83)
II	2 (4.17)
III	0 (0)
Anesthesia mode, n (%)	
General anesthesia	25 (52.08)
Lumbar anesthesia	23 (47.92)
Operation time, min (mean ± SD)	29.6 ± 2.49
Estimated blood loss, ml (mean ± SD)	34.9 ± 6.10
Painkiller demand, n (%)	
On POD 1	2 (4.17)
On POD 2	2 (4.17)
On ≥ POD 3	1 (2.08)
Complication, n (%)	
Haemorrhage	0 (0)
Urinary retention	1 (2.08)
Urinary tract infection	0 (0)
Incontinence	0 (0)
Other	0 (0)
Length of hospital stay, days (mean ± SD)	4.7 ± 2.23
Time to return to work/activities, days (mean ± SD)	2.2 ± 1.13
Follow-up period, months (mean ± SD)	11.3 ± 3.87

ASA, American Society of Anesthesiologists; POD, postoperative day.

**Table 4:** Perioperative and postoperative data of patients.

Variables	PRD1	POD30	POD90	POD180
CCF-IS (mean ± SD)	5.29 ± 5.06	5.50 ± 3.02	2.56 ± 2.92	1.29 ± 2.87
VAS-PS (mean ± SD)	3.33 ± 2.95	2.52 ± 2.22	1.19 ± 1.97	0.81 ± 2.28
QoLAF-QS, n (%)				
No impact (14)	9 (18.75)	18 (37.50)	37 (77.08)	45 (93.75)
Limited impact (15–28)	12 (25.00)	17 (35.42)	5 (10.42)	0 (0)
Medium impact (29–42)	12 (25.00)	7 (14.58)	3 (6.25)	1 (2.08)
High impact (43–56)	4 (8.33)	2 (4.17)	0 (0)	0 (0)
Bristol stool scale, n (%)				
Constipation (1–2)	3 (6.25)	1 (2.08)	0 (0)	0 (0)
Normal (3–5)	43 (89.58)	47 (97.92)	48 (100)	48 (100)
Watery (6–7)	2 (4.17)	0 (0)	0 (0)	0 (0)

PRD, preoperative day; POD, postoperative day; CCF-IS, Cleveland Clinic Florida incontinence score; VAS-PS, Visual Analog Scale pain score; QoLAF-QS, Quality of Life in Patients with Anal Fistula Questionnaire score.

At the 6-month follow-up, the average VAS-PS and CCF-IS score were 0.81 (SD 2.28; range, 0–10) and 1.29 (SD 2.87; range, 0–13), respectively (Table 4). No patient had anal incontinence.

## Discussion

The data from our single-center study demonstrate the feasibility and safety of the BEAS technique, with a favorable overall cure rate of 93.75% during the 6-month follow-up period. The overall in-hospital complication rate was 2.78%, and no other major complications were reported. The recurrence rate was 6.25%, all of which were successfully managed with timely surgical intervention. Furthermore, the BEAS technique appears to be superior to other techniques, such as the endorectal advancement flap, modified LIFT technique and autologous adipose-derived stem cell therapy, in terms of success rate and functional outcomes^20–22^.

HHAF is characterized by a highly located internal opening, complex fistula extension and a high recurrence rate. Sepsis typically propagates unilaterally or bilaterally from DPIS and DPAS along the midline and even extends to the gluteus maximus through the ischiorectal fossa. In light of the aforementioned characteristics of HHAF, we can summarize four basic principles for the treatment of HHAF. Firstly, the integrity of the external sphincter should be safeguarded. Secondly, the internal openings should be managed properly. Thirdly, the DPIS and DPAS should be occluded or attenuated. Finally, the purulent cavity should be fully drained.

Adequate drainage of DPIS and DPAS and keeping them open until the spaces close are of paramount importance. Due to the extensive involvement of the anal sphincter, adequate drainage of the open wound is bound to damage the anal sphincter, resulting in anal incontinence. In other words, a balance must be struck between adequate drainage and anal sphincter protection. Optimal treatment of the fistula and judicious management of the DPIS and DPAS while preserving anal function is the ideal outcome for the treatment of HHAF. Unfortunately, thus far, no procedure can simultaneously address all three issues. Furthermore, the internal opening of HHAF differs markedly from that of typical anal fistulas. It is often situated in the anterior and posterior midlines, and its height is often more than one-third that of the sphincter complex. If transanal internal sphincterotomy or the cutting seton procedure is employed to manage the internal openings, it will result in a large wound and take a long time to heal after surgery.

The findings of this study show that the BEAS procedure was effective and safe for the treatment of HHAF, with an overall healing rate of 93.75% and no incontinence. Three patients developed postoperative recurrence, one of whom was circular HHAF and two were posterior HHAFs. One patient with posterior HHAF was successfully treated with a second BEAS procedure. The advantages of the BEAS procedure are discussed below.

Firstly, the function and normal anatomical structure of the anal sphincter is preserved. In two-thirds of HHAF the primary fistula tract was high transsphincteric with a posterior primary opening and a circumferential spread in the DPIS and DPAS^23–26^. It is imperative to avoid the anal sphincter during operation, otherwise it will increase the risk of anal stricture or stricture formation post-operation. The protection of anal sphincter function, particularly EAS function, is one of the most critical determinants of long-term surgical success after surgery. Through two approaches (intersphincter approach and lateral-external-sphincteric approach), the EAS can be isolated and freed, which can be effectively safeguarded during the operation. The results of our study showed that the average CCF-IS on POD 180 (6 months after operation) was 1.29 (SD 2.87; range, 0–13), indicating that the EAS and the anal function were well preserved.

Secondly, minimally invasiveness and adequate drainage should be taken into account simultaneously. The internal opening should be completely closed. We shifted the musculocutaneous flap of the internal sphincter towards the distal side to displace the internal opening and fistula. The procedure of BEAS is quite similar to that of the transanal opening of intersphincteric space (TROPIS) procedure with a slight modification. Unlike TROPIS, the internal opening is processed by creating an intersphincteric space wound in BEAS and is closed by advancing the musculomucosal flap and EAS^27^. Then we sutured and fixed the EAS and the musculocutaneous flap with absorbable suture to close the DPIS and reduce the DPAS. The drainage of the DPIS and the DPAS is also a critical factor in the success of the operation. We should keep the LES approach open with a proper-length incision and confirm that the approach will lead to the cavity, facilitating the replacement of drainage after the operation. The results of our study showed that the average VAS-PS on POD 180 was 0.81 (SD 2.28; range, 0–4). The QoLAF-QS also showed that almost all the patients (93.75%) reached “no impact” on POD 180. Both the results of VAS-PS and QoLAF-QS suggest that smaller wound and higher quality of life can be achieved by using BEAS.

Thirdly, patients can return to their normal life earlier with less pain. The results showed that only two patients used painkillers within three days after surgery, and only one patient had urinary retention, which indicated that BEAS had less damage to the pelvic floor muscles. The results also showed that the “Normal” level of Bristol stool scale increased from 89.58 to 100% on POD 90. We believe that this may be due to BEAS improving the local inflammation of the anus in HHAF, thus allowing the patient to defecate normally.

Still, there are several limitations to our study. This was a single-center study with a small sample size, which limits the external validity and repeatability of our results. Further large controlled clinical trials are needed to determine the effectiveness and safety of this procedure. When evaluating the success of wound healing and wound closure, subjective judgments were made. Postoperative MRI, re-epithelialization of wound tissue and other objective indicators were lacking in our current study. Finally, this was a preliminary pilot observational cohort study that focus mainly on the short-term clinical outcomes for this technique. However, we will design large comparative studies or clinical controlled studies with long-term outcomes including 2-year follow-up period for relapse in the future to further validate the effectiveness and safety of this procedure.

In summary, BEAS is effective in terms of short-term results in the treatment of primary or recurrent HHAF. Although BEAS achieves a lower short-term recurrence rate, the long-term recurrence rate still needs to be investigated. This procedure is worthy of further promotion and application.

## Data Availability

The datasets we generated and analysed of this study are not made publicly available due to the regulations on the sensitive data of our institutional policies, but a de-identified data could be available from the first author (Dr. JY Zhu) upon reasonable request.

## Abbreviations

BEAS: The bared external anal sphincter
CCF-IS: Cleveland Clinic Florida incontinence score
DPAS: Deep postanal space
DPIS: Deep intersphincteric space
EAS: External anal sphincter
HHAF: High horseshoe anal fistula
IAS: Internal anal sphincter
IS Approach: Intersphincteric approach
LES Approach: Lateral-external-sphincteric approach
MRI: Magnetic resonance imaging
QoLAF-QS: Quality of life in patients with anal fistula questionnaire score
VAS-PS: Visual analog scale pain score
